# Retraction of: P-1834. Clinical profile and outcomes of hepatitis A virus–associated with severe acute liver injury in adults: A case study of Delhi, India

**DOI:** 10.1093/ofid/ofag307

**Published:** 2026-06-11

This is a retraction of Monirujjaman Biswas, P-1834. Clinical profile and outcomes of hepatitis A virus–associated with severe acute liver injury in adults: A case study of Delhi, India, *Open Forum Infectious Diseases*, Volume 13, Issue Supplement_1, January 2026, ofaf695.2003, https://doi.org/10.1093/ofid/ofaf695.2003.

The journal is retracting this IDWeek abstract because there is significant overlap between it and a previously published abstract without attribution:

Determinants, profile and outcomes of hepatitis A virus–associated severe acute liver injury in adults (https://doi.org/10.1007/s12664-024-01577-3). Additionally, the authorship of the publication cannot be verified.

